# Role of microRNAs in response to cadmium chloride in pancreatic ductal adenocarcinoma

**DOI:** 10.1007/s00204-021-03196-9

**Published:** 2021-12-14

**Authors:** Maria Mortoglou, Aleksandra Buha Djordjevic, Vladimir Djordjevic, Hunter Collins, Lauren York, Katherine Mani, Elizabeth Valle, David Wallace, Pinar Uysal-Onganer

**Affiliations:** 1 Cancer Research Group, School of Life Sciences, University of Westminster, London, W1W 6UW UK; 2 Department of Toxicology, University of Belgrade, 11000 Belgrade, Serbia; 3 First Surgical Clinic, Clinical Center of Serbia, Belgrade, Serbia; 4 College of Medicine and the Department of Pharmacology and Physiology, Oklahoma State University Center for Health Sciences, 1111 West 17th Street, Tulsa, OK 74107-1898 USA

**Keywords:** Pancreatic ductal adenocarcinoma, Non-coding RNAs, microRNAs, Cadmium, EMT, Apoptosis, LC50

## Abstract

Pancreatic ductal adenocarcinoma (PDAC) is one of the most fatal and aggressive malignancies with a 5-year survival rate less than 9%. Early detection is particularly difficult due to the lack of symptoms even in advanced stages. microRNAs (miRs/miRNAs) are small (~ 18–24 nucleotides), endogenous, non-coding RNAs, which are involved in the pathogenesis of several malignancies including PDAC. Alterations of miR expressions can lead to apoptosis, angiogenesis, and metastasis. The role of environmental pollutants such as cadmium (Cd) in PDAC has been suggested but not fully understood. This study underlines the role of miRs (miR-221, miR-155, miR-126) in response to cadmium chloride (CdCl_2_) in vitro. Lethal concentration (LC50) values for CdCl_2_ resulted in a toxicity series of AsPC-1 > HPNE > BxPC-3 > Panc-1 = Panc-10.5. Following the treatment with CdCl_2_, miR-221 and miR-155 were significantly overexpressed, whereas miR-126 was downregulated. An increase in epithelial–mesenchymal transition (EMT) via the dysregulation of mesenchymal markers such as Wnt-11, E-cadherin, Snail, and Zeb1 was also observed. Hence, this study has provided evidence to suggest that the environmental pollutant Cd can have a significant role in the development of PDAC, suggesting a significant correlation between miRs and Cd exposure during PDAC progression. Further studies are needed to investigate the precise role of miRs in PDAC progression as well as the role of Cd and other environmental pollutants.

## Introduction

PDAC is the deadliest and most prevalent pancreatic cancer (PCa) type, which accounts for 90% of PCa cases (Von Hoff et al. 2009; Hidalgo et al. 2015). PDAC can be characterized as a “silent killer” and presents the worst prognosis between all cancer types due to the widespread metastasis that patients appear at the time of diagnosis, which is usually in late stages of malignancy (Bortesi et al. 2011). Only a small portion of PDAC patients can benefit from chemotherapy, due to severe toxic effects of the current therapies (Abbruzzese 2008). Especially in advanced stages, the chemotherapeutics options are limited with gemcitabine being the first drug treatment with improvement in the median survival only by a few weeks (Scara et al. 2015; Xu et al. 2015). Carbohydrate antigen 19-9 (CA 19-9) is the most common diagnostic biomarker for PDAC in the last 30 years (Chan et al. 2014); nevertheless, CA 19-9 cannot be characterized as a PDAC-specific biomarker, especially for symptomless patients (Chan et al. 2014).

Interest in the role of Cd as a toxic metal ubiquitously present in the environment has risen during the last decades, especially with the reference to its possible role in various human diseases, especially carcinomas (Anđelković et al. 2021; Buha et al. 2018, 2019, 2020). Its possible role in PDAC has been recently reviewed by Buha et al. (2017) suggesting multiple mechanisms responsible for Cd actions leading to the development of PDAC. The three predominant toxic mechanisms proposed are the alterations in the redox status of the cell, changes in the apoptotic pathways, and epigenetic changes. Further research which was a synergy of human observational, experimental, and in vitro studies showed Cd deposition in pancreatic tissue and revealed Cd-induced disturbances in intrinsic pathway of apoptotic activity and elevated oxidative stress in PDAC cells (Djordjevic et al. 2019). Further studies conducted on PDAC cell lines indicated that the mitochondria may be a site of action for Cd in promoting tumour development (Wallace et al. 2019). Such findings clearly point towards Cd exposure as an important risk factor for PDAC development.

Preliminary studies suggested a correlation between aberrant expression levels of numerous miRs and PDAC (Yu et al. 2012). miRs, small (18–28 nucleotides-long), endogenous, non-coding, evolutionary conserved, single-stranded RNA molecules, shown to moderate gene expression at the post-transcriptional level through the binding to the complementary sequences of their target messenger RNAs (mRNAs) at the 3′ untranslated regions (3′ UTRs) (Bartel 2004). Based on the interactions between the 3′ UTRs of mRNAs, miRs can control the expression levels of several genes, while they can also regulate a number of cell signalling pathways, which are related to several malignancies (Lin and Gregory 2015; Meltzer 2005; Słotwiński et al. 2018). Consequently, alterations in miRs expression can have as an outcome, apoptosis, angiogenesis, and metastasis (Amirkhah et at. 2015).

miR-221 is one of the most oncogenic miRs in PDAC alongside miR-21 (Bloomston et al. 2007; Uysal-Onganer et al. 2021; Yang et al. 2016; Zhang et al. 2008), while the overexpression of miR-221 has been correlated to a number of malignancies, such as hepatocellular carcinoma, prostate adenocarcinoma, and colorectal carcinoma (Liu et al. 2014; Tao et al. 2014; Yau et al. 2014; Zheng et al. 2014). Enhanced expression levels of miR-221 are closely associated with platelet-derived growth factor (PDGF)-mediated epidermal–mesenchymal transition phenotype, migration, metastasis, and uncontrolled proliferation of PDAC cells through the inhibition of both mitogen-activated protein kinase (MAPK) and transformation of growth factor β (TGF-β) signalling pathways (Masamune et al. 2013; Su et al. 2013; Mortoglou et al. 2021a, b). EMT can be characterized as a key component of the metastatic cascade, which includes the repression of E-cadherin and the activation of genes related to motility and invasion (Kalluri and Weinberg 2009). Therefore, the examination of the most commonly expressed miRs with their related signalling pathways and their associated target genes is crucial for a better understanding of PDAC pathophysiology. Furthermore, upregulation in the expression of miR-155 has been also found in PDAC tissue samples (Papaconstantinou et al. 2013). Elevated levels in the expression of miR-155 can result in poor survival in PDAC patients due to the development of fibrogenesis, through TGF-β (Greither et al. 2010; Mortoglou et al 2021a,b)*,* while overexpression of this miR has been also associated with an increased progression from pancreatic intraepithelial neoplasia 2 (PanIN-2) to PanIN-3 (Ryan et al. 2014). On the other hand, miR-126 is a tumour suppressor miR, which has been linked to PDAC progression, through the post-transcriptional upregulation of *KRAS* (Jiao et al. 2012) and *HER2* (Garajová et al. 2014). *HER2* overexpression is observed in more than 30% of PDAC cases (Komoto et al. 2009; Mortoglou et al. 2021a,b).

In metastatic process, EMT is responsible for the loss of epithelial cell polarity and cell–cell adhesion (Brabletz et al. 2018; Ye et al. 2017), while studies have shown that miRs are linked to the moderation of EMT, stem cell-like differentiation, invasiveness, migration, and proliferation (Piasecka et al. 2018; Mortoglou et al. 2021a, b). Specifically, EMT is involved in increased tumorigenesis, invasiveness, and metastatic predisposition of several solid tumours including prostate, lung, liver, pancreatic, and breast cancers (Hugo et al. 2007; Lee et al. 2006; Ribatti et al. 2020). In numerous malignancies, EMT can be induced by hypoxia, growth factors regulated by tumour microenvironment, stroma crosstalk, metabolic changes, and innate and adaptive immune responses (Roche 2018).

Wnt-11 is one of the noncanonical Wnt family members, which are activated during carcinogenesis and correlated to a poor prognosis of various cancer types including PDAC (Uysal-Onganer et al. 2010; Arisan et al. 2020; Guo and Wang 2009). During EMT, overexpression of the mesenchymal marker Snail and the decrease of the epithelial marker E-cadherin has been noticed (Lin et al. 2010; Nakamura and Tokura 2011). Particularly, E-cadherin is a protein, which is necessary for the normal epithelial cell maintenance, whereas transcriptional factors including Zeb1 and Snail are highly expressed through the binding in E-cadherin promoter (Felipe Lima et al. 2016; Lamouille et al. 2014; Seton-Rogers 2016; Singh and Settleman 2010). Therefore, transcription factors such as Snail and Zeb1 are key players of EMT (Stemmler et al. 2019). Moreover, EMT is regulated via molecular pathways, which are linked to both oncogenic and tumour suppressor non-coding RNAs, chromatin remodelling, epigenetic and posttranslational modifications, alternative splicing events, and protein stability (De Craene and Berx 2013; Fedele et al. 2017). Wnt-11 overexpression has been observed in PDAC tissues compared to normal adjacent tissues and is associated with tumour-node-metastasis (TNM) staging (Wang et al. 2018a, b). Especially, higher levels of Wnt-11 are correlated to stages II, III, and IV (Wang et al. 2016). On the other hand, Zeb1 upregulation is correlated to advanced PDAC stages and poor malignancy outcome (Arumugam et al. 2009; Buck et al. 2007; Maier et al. 2010). Zeb1 is also responsible not only for the acquisition of an EMT phenotype but also for migration and invasion in response to nuclear factor kappa-light-chain-enhancer of activated B cells (NF-κβ) signalling in PDAC cells (Maier et al. 2010). Furthermore, downregulation of E-cadherin is associated with poor prognosis and differentiation in PDAC (Iacobuzio-Donahue et al. 2009; Winter et al. 2008), while elevated expression levels of Snail have been found to be related to 80% of PDAC cases (Hotz et al. 2007). Besides, Snail overexpression is connected to reduced E-cadherin expression, higher tumour grade, and poorly differentiated PDAC cell lines (Hotz et al. 2007).

Recently, mounting evidence has shown that heavy metals, including Cd, might exert their toxicity through miRs (Wallace et al. 2020). Hence, this study aimed to investigate miR expressions in response to Cd in metastatic PDAC cells. Subsequently, due to the fact that EMT markers could be proven a novel target for anticancer therapy, we also examined the expression levels of Wnt-11, E-cadherin, Snail, and Zeb1 following Cd exposure in PDAC in vitro.

## Methodology

### Pancreatic cell cultures and cadmium treatment

The cell lines are obtained from the American Type Culture Collection (ATCC, Manassas, VA). Control pancreas cells [hTERT-HPNE (“human pancreatic Nestin-expressing” cells or HPNE; ATCC^®^ CRL-4023™, control pancreatic cells)] and tumour [AsPC-1 (ATCC^®^ CRL-1682™, pancreatic tumour cells), Panc-1 (ATCC #CRL-1469™, Pancreas ductal epithelioid carcinoma), Panc-10.05 (ATCC #CRL-2547™, Pancreatic epithelial adenocarcinoma), and BxPC-3 (ATCC #CRL-1687™, Pancreatic adenocarcinoma)] were grown and maintained as described in the ATCC-suggested protocols. Unless otherwise specified, cells were grown in their defined optimum growth media. For a parallel set of LC50 assays, cells were grown in a minimal media of MEM plus 1% foetal bovine serum (FBS). The cells were split in 6-well plates at 3–5 day intervals depending on confluence and cells were grown to 80% confluency in preparation for 14 days treatment with cadmium chloride (CdCl2; 50 μM), based on our previously published data (Djordjevic et al 2019).

### Chemicals and antibodies

CdCl_2_, sterile dimethylsulfoxide, sterile phosphate-buffered saline, and molecular grade water were purchased as the highest grade required from Sigma-Aldrich (St. Louis, MO USA), Fisher Scientific (Houston, TX USA), and Pierce Biotechnology (Rockford, IL USA). ELISA kits were obtained from Pierce Biotechnology (Rockford, IL USA). Antibodies were obtained from R&D Systems Inc., (Minneapolis, MN USA), Cell Signaling Technology, Inc. (Danvers, MA USA), and Thermo-Fisher Scientific (Waltham, MA USA). Media for each cell line were obtained from Corning Life Sciences (Tewksbury, MA USA). Media supplements were purchased from Sigma-Aldrich (St. Louis, MO USA), and these included penicillin/streptomycin, glucose, glutamine, and sodium bicarbonate. Foetal bovine serum, triple 0.1 μm filtered, was purchased from Atlanta Biologicals through R&D Systems (Minneapolis, MN USA).

### LC50 assays to determine cadmium toxicity after 48 h exposure

Each of the five cell lines were plated at an initial density of 20–50,000 cells/well and allowed to adhere for 24 h prior to the addition of CdCl_2_. Cells were incubated for 48 h with increasing concentrations of CdCl_2_ (12 concentrations, 0—1 mM). After exposure, MTT (3-(4,5-dimethylthiazol-2-yl)-2,5-diphenyltetrazolium bromide) stock solutions (11 mM in sterile PBS) were diluted in-well by the addition of 10 μL of MTT stock to each well (1.1 mM final concentration). Plates were returned to the incubator (37 °C/5% CO_2_) for 4 h. After incubation, 50 μL of DMSO was added to each well to solubilize MTT crystals, and the plates were returned to the incubator for 30 min. Absorbance was measured using a Bio-Tek plate reader at 540 nm and is directly proportional to the number of live cells.

### ELISA assays and protein detection

The human β-catenin kit was purchased from MyBioSource, Inc. (MBS266009; San Diego, CA USA), and was designed for measuring β-catenin from human-derived cells and cell supernatants. Cell preparation and assay protocols were essentially as described in the manufacturer’s protocol. The quantity of β-catenin in the sample extracts was extrapolated from a standard curve using β-catenin concentrations of 0 pg/mL to 1000 pg/mL. Cell lysates were prepared after exposure to Cd for 48 h by trypsinization, centrifugation, and washing (3x) in PBS. The resulting cell suspension was subjected to three freeze–thaw cycles at − 20 °C. The suspension was centrifuged at 1000 ×*g* (or 3,000 rpm) for 15 min at + 2–4 °C to remove cellular debris. The final supernatant is ready for assay, or can be stored at − 80 °C until use. The amount of β-catenin present was quantified using a biotinylated β-catenin antibody (1:100 dilution).

In-Cell ELISA protocols for the remaining assays were based on the Pierce Biotechnology kit with the antibody either being provided in the kit, or purchased separately. The assay procedures are the same for each. Cells were exposed to 1 μM CdCl_2_ for 48 h in a 37 °C/5% CO_2_ incubator. After incubation, media are removed and 4% formaldehyde is added to each well to fix the cells. Cells are washed, permeabilized, quenched, and blocked in sequential fashion. The primary antibody is added, and the plated sealed and stored at + 4 °C overnight. The dilutions of each primary antibody were as follows: phospho-Akt (AF887, R&D Systems; 1:2000); phosphatase and tensin homolog (PTEN) (AF847, R&D Systems; 1:1000); Poly (ADP-ribose) polymerase (PARP) (1861790, Thermo-Fisher; 1:1000); Forkhead box protein O1 (FOXO1) (2880S, Cell Signalling; 1:1,000); and tumour protein 53 (p53) (1861777, Thermo-Fisher; 1:1000). Following incubation overnight at + 4 °C, cells are removed, washed and a TMB solution (3,3′, 5,5;-tetramethylbenzidine) is added. TMB is a chromogen that can be used in place of horseradish peroxidase (HRP). Absorbance is measured using a Bio-Tek^®^ plate reader and Gen5 software at 450 nm. To standardize the protein expression data, Janus Green whole-cell stain was used to account for potential differences in cell number between wells. Janus Green staining is measured by absorbance at 615 nm. Dividing the absorbance at 450 nm by the absorbance at 615 nm yields a relative protein expression.

### Caspase 3/7 kinetic assays

Caspase 3/7 activity (measurement of apoptosis) was determined using the Apo-One™ Homogeneous caspase-3/7 assay (Promega, Madison WI). Both Caspase 3 and 7 convert the non-fluorescent substrate rhodamine 110, bis-(N-CBZL-aspartyl-L-glutamyl-L-valyl-L-aspartic acid amide; Z-DEVD-R110), by removing the DEVD peptides. Removal of the DEVD peptide results in the rhodamine 110 leaving group being excited at a wavelength of 499 nm with an emission wavelength of 521 nm. The fluorescence generated is directly proportional to the amount of caspase 3 and 7 present. Cells were plated as described above and the treatment was started with the addition of 1 μM CdCl_2_. Plates are returned to the incubator for 48 h (37 °C/5% CO_2_). After 48 h exposure, caspase 3/7 activity was determined by the addition of 100 µL (1:1 with media) of the caspase 3/7 substrate/buffer mix. Plates were wrapped in foil and returned to the incubator (37 °C/5% CO_2_) for 1 h. Emitted fluorescence was measured using a Bio-Tek^®^ plate reader and Gen5 software at 485_ex_/530_em_. Additional readings were taken at 3 h and 6 h for kinetic assessment of caspase 3/7 activity.

### RNA extraction and quantitative real-time PCR

miR expression levels were assessed in non-treated and treated with CdCl_2_ Panc-1 and MiaPaCa-2 PDAC cell lines. Panc-1 and MiaPaCa-2 cell lines were processed for RNA isolation, followed by cDNA translation and assessment for the relative expression of miR-155, miR-221, and miR-126. RNA extraction was carried out using Trizol (Sigma, U.K.), while RNA concentration and purity were measured by NanoDrop Spectrophotometry at 260 nm and 280 nm absorbance. Reverse transcription of RNA to cDNA was carried out using the miRCURYâLNAâ RT Kit (Qiagen, U.K.). U6-snRNA was used as reference RNA to normalise miR expression levels. The miRCURYâ LNAâ miRNA SYBRâ Green (Qiagen, U.K.) was used in conjunction with MystiCq microRNA qPCR primers for miR-155 (hsa-miR-155-5p), miR-126 (hsa-miR-126-5p), and miR-221 (hsa-miR-221-5p), which were all obtained from Sigma (U.K.). The sequences for U6-snRNA primers were U6 forward, 5′-GCTTCGGCAGCACATATACTAAAAT-3′ and reverse 5′-CGCTTCACGAATTTGCGTGTCAT-3′ for both. The conditions for thermocycling were: heat activation at 95 °C/2 min, followed by 40 cycles at denaturation at 95 °C/10 s and combined annealing/extension at 56 °C/60 s. The miR-155, miR-126, and miR-221 expression levels were normalised to that of U6 using the 2^^ΔΔCT^ method. Normal cDNAs were generated using qScriptä cDNA Supermix (Quantabio, UK) with incubations at 22 °C for 5 min, 42 °C for 30 min, and 85 °C for 5 min, and the following genes were examined: Wnt-11, E-cadherin, Snail, and Zeb1. PrecisionâPlus qPCR Master Mix (Primer Design, UK) was used for RT-qPCR synthesis with the following thermocycling conditions for 40 cycles; 95 °C for 2 min, 95 °C for 10 s, and 95 °C for 60 s. Relative levels of mRNA expression were calculated using the comparative CT/2^−ΔΔCT^ method with RNA polymerase II (RPII) as the reference gene.

### Statistical analysis

GraphPad Prism version 9.01 (GraphPad Software, San Diego, U.S.A.) was used for statistical analysis and preparation of graphs. Paired *t* tests were used to assess the expression levels of selected miRs and proteins compared with the controls. Experiments were carried out in triplicates for miRs and protein analysis. All non-miRNA data were analyzed using GraphPad Prism (v 9.1–9.3; San Diego, CA). Analyses include one-way ANOVA with the specialized Dunnett’s test for post hoc comparison to control values. Data analysis was accomplished using a two-way (Cell type × treatment) or three-way (Cell type × treatment × protein) ANOVA with Tukey’s test adjusted for multiple comparisons or Sidak’s post hoc comparison test, as listed. LC50 values were determined by fitting the data to a nonlinear curve fitting, log concentration × response (three-parameter) single-site model, to yield a sigmoid inhibition curve. All data are expressed as the mean ± standard deviation (SD) of 4–8 assays performed in duplicate or triplicate were indicated. To test for data normality, the D’Agostino–Pearson omnibus test was performed. When the test yielded significant results, the data were deemed to be not normally distributed. For data that violated the assumption of normal distribution, the nonparametric Kruskal–Wallis test was performed, followed by a post hoc comparison using the corrected Dunn’s test. Significance is set at *α* = 0.05.

## Results

In summary, we determined the LC50 value for CdCl_2_ in multiple PDAC cell lines. One concern that needed to be addressed was the ability of complete growth media to potentially interfere with the action of the toxic metal. Parallel assays were performed, one in ATCC described growth media and another set in a minimal growth media with only 1% FBS supplementation and no phenol. Interestingly, we found that there was no media effect in AsPC-1 cells, and the HPNE cells seemed to be more resistant to the lethal effect of CdCl_2_. The remaining cell lines all responded with increased sensitivity to the lethal effects of CdCl_2_ significantly lowering the LC50 values in the presence of minimal media. When examining proteins associated with apoptotic processes, there were few CdCl_2_-related effects, with the many of the differences being related to the cell line, and differences in either basal activity or expression. It may be of interest that in studies examining the expression of phospho-Akt and β-catenin, tumour cells appeared to respond in the opposite direction as HPNE control cells suggesting the potential for differential responses. We further assessed the expression levels of specific miRs in response to CdCl_2_ exposure. The current study found that miR-221 and miR-155 were upregulated in metastatic PDAC cell lines treated with CdCl_2_, while miR-126 was downregulated. We have also noted that CdCl_2_ altered the expression levels of EMT markers such as Wnt-11, E-cadherin, Snail, and Zeb1. Particularly, Wnt-11, Snail and Zeb1 were considerably upregulated following CdCl_2_ exposure, while E-cadherin expression level was decreased in PDAC cell lines treated with CdCl_2_ compared to non-treated cells.

### LC50 assays to determine cadmium toxicity after 48 h exposure

#### CdCl_2_ LC50 analysis in PDAC cells

**Fig. 1:**
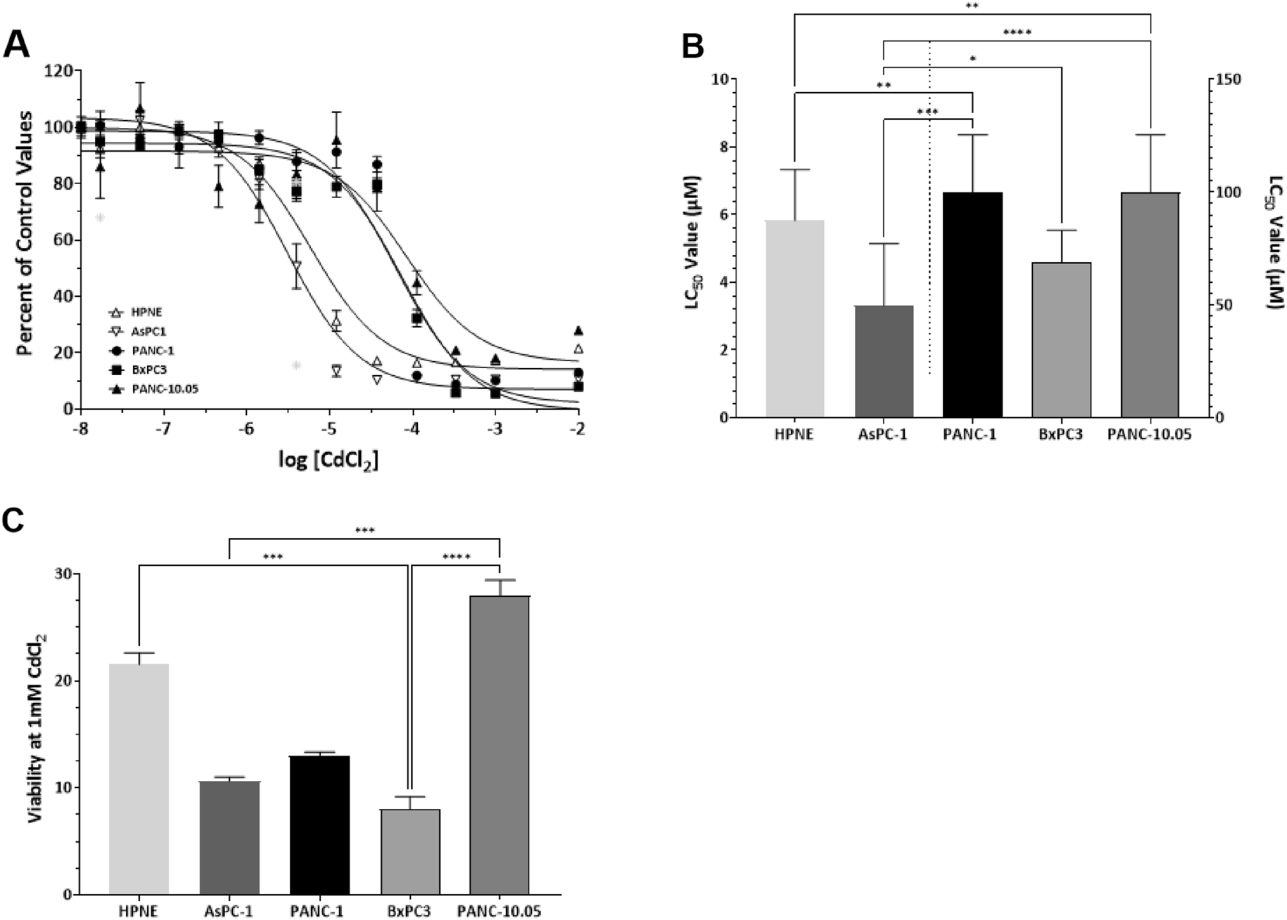
Comparison of LC50 values in PDAC cells grown in optimum growth media. Exposure to increasing concentrations of CdCl_2_ resulted in increased lethality in all PDAC cell lines. Cells were plated according to what is outlined in “Methods” and exposed to CdCl_2_ for 48 h. Live cell numbers were determined by the MTT assay and the data were fit to a nonlinear curve fitting program using a single-site, three-parameter model. The best-fit curve for each of the assays is represented in (**A**). Individual curves were analyzed and LC50 values obtained to compare group LC50 values (**B**). When comparing LC50 values, the Panc-1, BxPC-3, and Panc-`0.05 cells are represented on the right axis. Maximum lethality in the presence of CdCl_2_ occurred at concentrations greater than 1 mM. Therefore, (**C**) represents the maximum lethality of 1 mM CdCl_2_ in each of the PDAC cell lines. Kruskal–Wallis analysis was used to assess differences in LC50 values between PDAC cell lines and Dunn’s test for multiple comparisons. Data represent a mean of eight (*n* = 8) assays performed in duplicate (mean ± SD). Exact *p* values are indicated (* *p* ≤ 0.05; ** *p* ≤ 0.01; *** *p* ≤ 0.001; **** *p* ≤ 0.0001)

Initial LC50 analysis to determine the lethality thresholds was performed in optimum growth media which provides the cells with the required nutrients, and protectants, for optimum growth. Each of the cell lines were incubated with their optimum growth media as defined by ATCC. Cells were allowed to adhere for 24 h and the exposure was started by the addition of increasing concentrations of CdCl_2_ and the cells were returned to the incubator (37 °C/5% CO_2_) for 48 h. We observed a reduction in cell viability with increasing concentrations of CdCl_2_ (Fig. 1A). The shapes of the curves were best fit to a single-site model (three-parameter model). Between cell lines, there was a clear shift to the left for HPNE and AsPC-1 cells (lower LC50 values), whereas Panc-1, BxPC-3, and Panc-10.5 cells were shifted 10–15-fold to the right (Fig. 1B). Kruskal–Wallis analysis revealed a significant difference in LC50 values between cell lines H(5) = 31.97; *p* < 0.0001. Using Dunn’s test for multiple comparisons, control HPNE LC50 values were significantly lower than Panc-1 (*p* < 0.01) and Panc-10.05 (*p* < 0.01). LC50 values for AsPC-1 cells were significantly lower compared to all other cancer lines, Panc-1 (*p* < 0.001), BxPC-3 (*p* < 0.05), and Panc-10.05 (*p* < 0.0001). The comparisons between LC50 are also in Table1 where the assay media comparison is examined (Table 1). A lethality series can be obtained based on LC50 values, with AsPC-1 ≤ HPNE < BxPC-3 < Panc-1 = Panc-10.05. The maximum lethality at 1 mM CdCl_2_ was compared across cell lines, and we observed significant differences across cell line H(5) = 37.22; *p* < 0.0001 (Fig. 1C). PDAC cells were more sensitive to the actions of CdCl_2_ with AsPC-1, Panc-1, and BxPC-3 cells exhibiting almost 90% lethality. Control HPNE cells exhibited about 80% lethality at 1 mM CdCl_2_, but Panc-10.05 cells were the most resistant with only about 70% lethality.

**Table 1:** Comparison of LC50 values in ATCC-defined optimum growth media compared to minimal MEM media with 1% FBS

	Cadmium LC_50_ values (μM) [mean ± SD)	
Cell type	Optimum growth media	Minimal media (MEM + 1% FBS)
HPNE	5.8 ± 1.5	36.8** ± 5.4
AsPC-1	3.3 ± 1.8	6.0 ± 1.9
Panc-1	104.0 ± 24.7	17.7 *** ± 3.2
BxPC3	69.2 ± 14.0	12.7*** ± 7.2
Panc-10.05	104.1 ± 24.5	8.9*** ± 1.7

Dunn’s test for multiple comparisons was used to assess control HPNE LC50 values compared with the selected PDAC cell lines (AsPC-1, Panc-1, BxPC-3, and Panc-10.05). Exact *p* values are indicated (* *p* ≤ 0.05; ** *p* ≤ 0.01; *** *p* ≤ 0.001; **** *p* ≤ 0.0001)

#### Effect of minimal media on CdCl_2_ LC50 values

**Fig. 2:**
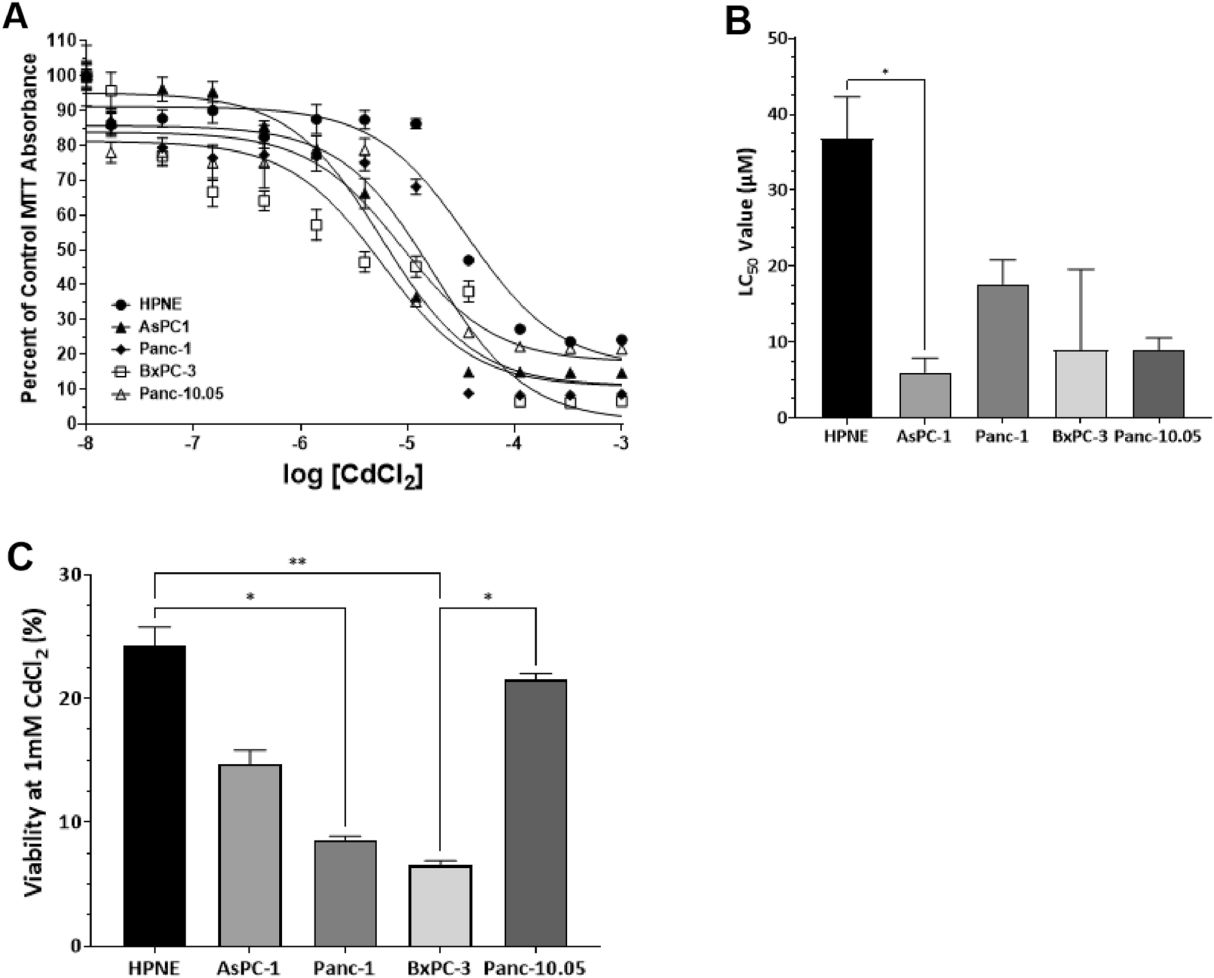
Comparison of LC50 values in PDAC cells exposed to CdCl_2_ in minimal (MEM + 1% FBS) media. Exposure to increasing concentrations of CdCl_2_ resulted in increased lethality in all PDAC cell lines. Cells were plated according to what is outlined in “Methods” and exposed to CdCl_2_ for 48 h. Live cell numbers were determined by the MTT assay and the data were fit to a nonlinear curve fitting program using a single-site, three-parameter model. The best-fit curve for each of the assays is represented in (**A**). Individual curves were analyzed and LC50 values obtained to compare group LC50 values (**B**). Maximum lethality in the presence of CdCl_2_ occurred at concentrations greater than 1 mM. Therefore, (**C**) represents the maximum lethality of 1 mM CdCl_2_ in each of the PDAC cell lines. Kruskal–Wallis analysis was used to assess differences in LC50 values between PDAC cell lines and Dunn’s test for multiple comparisons. The data represent a mean of eight (*n* = 4) assays performed in duplicate (mean ± SD). Exact *p* values are indicated (* *p* ≤ 0.05; ** *p* ≤ 0.01; *** *p* ≤ 0.001; **** *p* ≤ 0.0001)

A concern when performing toxicity studies in cell culture is trying to mimic the normal cellular environment as close to normal as possible. The use of optimum growth media provides the cells with the required nutrients, and protectants, for optimum growth. However, it is possible that some of the ingredients of the media can reduce the toxicity observed with environmental toxicants. Our parallel set of assays in a minimal media (MEM + 1% FBS) with reduced glutamine supplementation, and without phenol, could provide a different environment that would promote toxicity. We observed a reduction in cell viability with increasing concentrations of CdCl_2_ (Fig. 2A). The shapes of the curves were similar to what was observed in optimum growth media, but the potency of CdCl_2_ significantly shifted (Fig. 2B). Kruskal–Wallis analysis revealed a significant difference in LC50 values between cell lines H(5) = 13.54; *p* = 0.0089. Using Dunn’s test for multiple comparisons, the only groups that were significantly different were HPNE and AsPC-1 (*p* = 0.0125) cell lines. Interestingly, the potency order between the cell lines was significantly different compared to what was observed in the optimum growth media (Table 1). The maximum lethality at 1 mM CdCl_2_ was compared across cell lines, and we observed significant differences across cell line H(5) = 18.29; *p* = 0.0011 (Fig. 2C). PDAC cells were more sensitive to the actions of CdCl_2_ with both Panc-1 and BxPC-3 cells exhibiting > 90% lethality. Control HPNE cells were the most resistant with about 75% lethality at 1 mM CdCl_2_.

### Effect of Cd exposure on caspase 3/7 activity

**Fig. 3:**
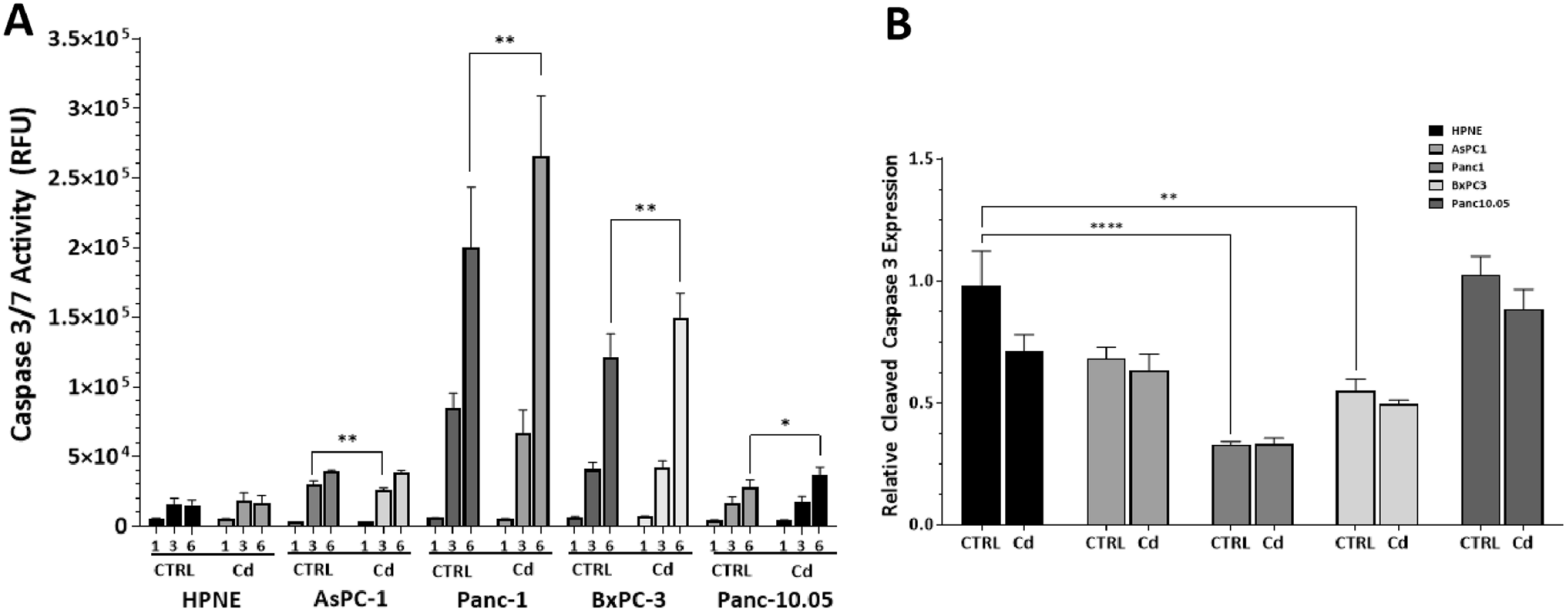
Changes in Caspase 3/7 activity at 1, 3, and 6 h (**A**) and the expression of cleaved caspase 3 (**B**) following 48 h CdCl_2_ exposure. After 48 h exposure, caspase 3/7 activity was determined using the Apo-One™ Homogeneous caspase-3/7 assay. The fluorescence generated in this kit is directly proportional to the amount of caspase 3 and 7 present. Fluorescence was measured at 1, 3, and 6 h after the addition of the non-fluorescent substrate (**A**). Emitted fluorescence of the product was measured using a Bio-Tek ® plate reader and Gen5 software at 499_ex_/521_em_. The amount of cleaved caspase 3 expressed was measured with a modification of the Pierce assay kit. After 48 h exposure, media were removed and 4% formaldehyde added to fix the cells in the wells. Primary antibody for cleaved caspase (Invitrogen; PA5-23921) was diluted 1:1,000, added to the fixed cells, and stored at + 4 °C overnight. After TMB administration, absorbance was measured at 450 nm. Cells were stained with Janus Green to account for differences in cell number between wells and absorbance read at 615 nm. Dividing the absorbance at 450 nm by the absorbance at 615 nm yields a relative protein expression. Two-way ANOVA with Tukey’s post hoc analysis was used to examine the expression levels of Caspase 3/7 activity in the selected PDAC cell lines following exposure to CdCl_2_. The data represent a mean of four (*n* = 4) assays performed in duplicate (mean ± SD). Exact *p* values are indicated (* *p* ≤ 0.05; ** *p* ≤ 0.01; *** *p* ≤ 0.001; **** *p* ≤ 0.0001)

We examined the activity of caspase-3, a critical executioner caspase, after exposure to CdCl_2_ for 48 h. The rationale for examining caspase-3 activity was that they are pivotal for apoptosis and cell death. Normally, caspase-3 is found in a procaspase form, which is inactive. To activate, either extrinsic factors, death receptor stimulation, or intrinsic, mitochondrial damage, will trigger the activation of caspase-3. Time-course analysis was performed and the ability of caspase-3 to cleave Z-DEVD-NH from rhodamine 110, resulting in a fluorescent signal that is proportional to the amount of caspase-3 present. Caspase-7 also recognized the DEVD bond, so the data are expressed as caspase-3/7 activity. We measured the activity of caspase-3/7 at for 1, 3, or 6 h after a 48 h exposure to CdCl_2_ (Fig. 3A).

Expression of cleaved caspase-3 (active caspase) was determined by ELISA (Fig. 3B). There was a significant effect dependent on cell line type for cleaved caspase-3 expression (F_4,30_ = 25.87; *p* < 0.0001). We also observed a significant effect related to CdCl_2_ exposure across all cell lines (F_1,30_ = 5.288; *p* = 0.028). The trend was for CdCl_2_ exposure to cause a reduction in expression, with basal expression of cleaved caspase 3 being significantly lower in Panc-1 (*p* < 0.0001) and BxPC-3 (*p* = 0.0044) cells compared to control HPNE cells.

### Effect of Cd exposure on the expression of proteins associated with apoptosis

**Fig. 4:**
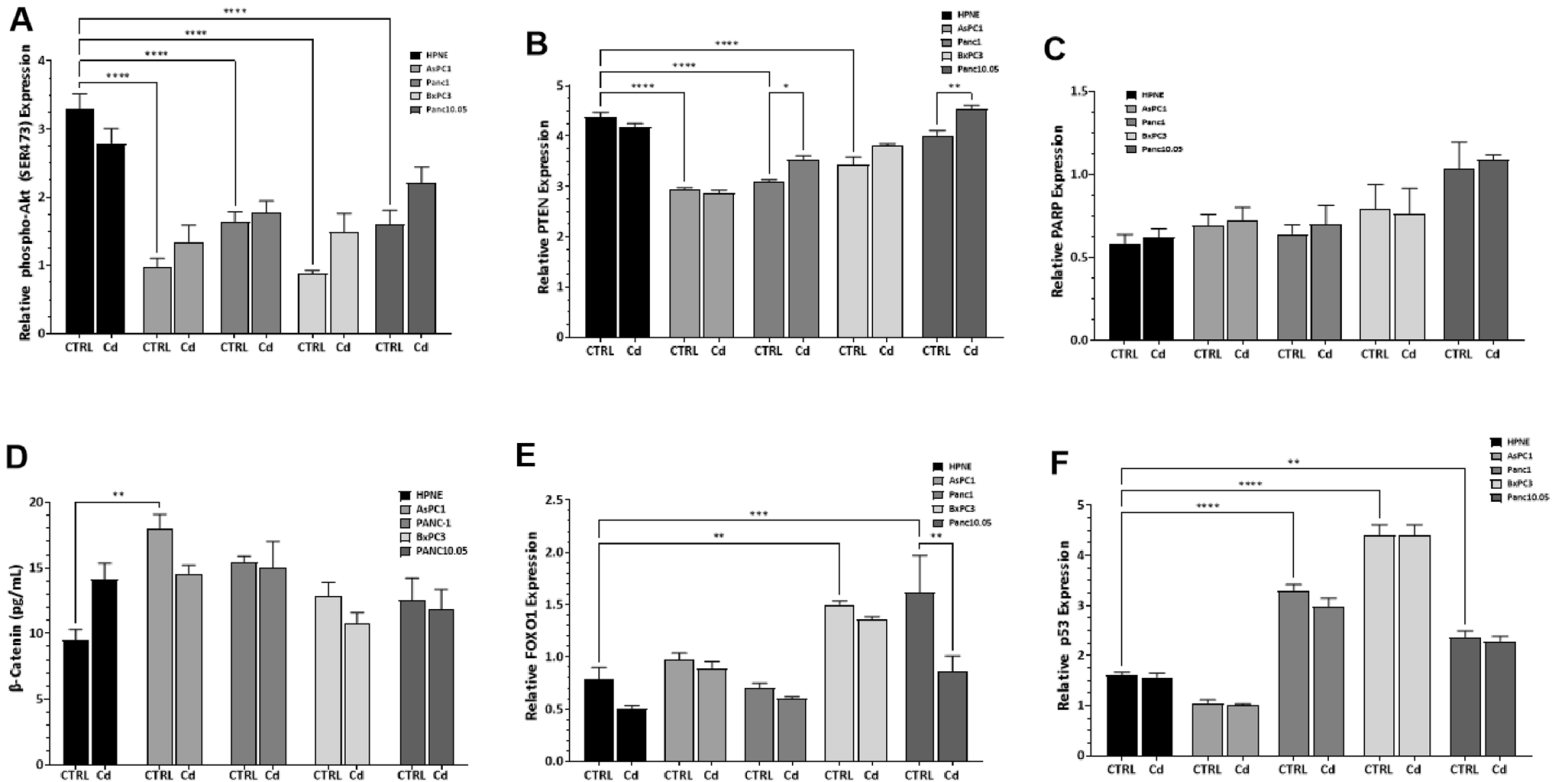
Changes in protein expression following 48 h exposure to CdCl_2_. Proteins associated with cell function and apoptosis were selected and the expression of phospho-Akt (**A**), PTEN (**B**), PARP (**C**), β-catenin (**D**), FOXO1 (**E**), and total p53 (**F**) were measured. The amount of cleaved caspase 3 expressed was measured with a modification of the Pierce assay kit as described in ‘Methods’. After 48 h exposure, media were removed and 4% formaldehyde added to fix the cells in the wells. Primary antibodies were diluted 1:500—1:2,000, added to the fixed cells, and stored at + 4 °C overnight. After TMB administration, absorbance was measured at 450 nm. Cells were stained with Janus Green to account for differences in cell number between wells and the absorbance read at 615 nm. Dividing the absorbance at 450 nm by the absorbance at 615 nm yields a relative protein expression. Three-way ANOVA followed by Tukey’s multiple comparison post hoc test was used to assess the expression levels of PTEN, PARP, β-catenin, FOXO1, and p53 following 48 h exposure to CdCl_2_ compared with the non-treated PDAC cells. The data represent a mean of four (*n* = 4) assays performed in duplicate (mean ± SD). For the β-catenin assays, the data represent a mean of three (*n* = 3) assays performed in duplicate (mean ± SD). Exact *p* values are indicated (* *p* ≤ 0.05; ** *p* ≤ 0.01; *** *p* ≤ 0.001; **** *p* ≤ 0.0001)

We examined the expression of several proteins associated with apoptosis, many of which are implicated in the progression of PDAC. There was a consistent significant main effect across each of the cell lines regardless of the protein that is being examined. Data analysis was carried out using a three-way ANOVA followed by Tukey’s multiple comparison post hoc test. Expression of phospho-Akt (Fig. 4A) was clearly different between cell lines (F_4,30_ = 29.84; *p* < 0.0001), Post hoc analysis comparison test demonstrated that control HPNE cell expression of phospho-Akt was significantly higher than the control group in each of the tumour cell lines. PTEN (Fig. 4B) expression was significantly affected by 48 h exposure to CdCl_2_ with a significant main effect of both treatment (F_1,30_ = 17.03; *p* = 0.0003) and cell line (F_4,30_ = 106.5; *p* < 0.0001). There was also a significant cross-over interaction (F_4,30_ = 8.12; *p* = 0.0001). The cross-over interaction is evident by the slight decline in PTEN expression in the control HPNE cells following CdCl_2_ exposure, compared to slight increases in PTEN expression across the tumour cell lines. Expression of PARP (Fig. 4C) was only slightly affected by CdCl_2_ exposure. There was a significant effect of cell line type on PARP expression (F_4,30_ = 6.14; *p* = 0.001). There were no differences between control and CdCl_2_ exposure in any cell line.

For the quantification of β-catenin, we were able to directly measure the amount of β-catenin in the cell lysates (Fig. 4D). In control HPNE cells, CdCl_2_ exposure for 48 h resulted in a 50% increase in β-catenin, but this difference was not significant. We observed a significant effect of cell line type on β-catenin content (F_4,20_ = 5.94; *p* = 0.0026) with a significant cross-over interaction with treatment (F_4,20_ = 3.1; *p* = 0.039)*.* Generally, tumour cells exhibited a higher basal level of β-catenin, with AsPC-1 cells demonstrating nearly double the basal level of β-catenin compared to control HPNE cells (*p* = 0.0028). We examined the effect of CdCl_2_ exposure on FOXO1 expression (Fig. 4E) due to the involvement of the FOXO proteins and sirtuin 1 (SIRT1) in glucose handling, development of diabetes, and potential role in the development of PDAC. There was a significant (F_4,29_ = 21.54; *p* < 0.0001) effect of cell line type on the expression of FOXO1 with both BxPC-3 and Panc-10.05 demonstrating a twofold increase compared to control HPNE cells. There was a significant effect of treatment (F_1,29_ = 15.88; *p* = 0.0004) across cell lines with CdCl_2_ exposure reducing FOXO1 expression, but this change only reached significance in the Panc-10.05 cell line. Finally, we measured the expression of p53 (total) in PDAC cells (Fig. 4F) and observed a significant effect of cell line type on the expression (F_4,30_ = 218.0; *p* < 0.0001). Exposure to CdCl_2_ had no effect on p53 expression across each of the cell lines. Basal level of p53 expression was significantly elevated compared to HPNE control cells in Panc-1 (*p* < 0.0001), BxPC-3 (*p* < 0.0001), and Panc-10.05 (*p* < 0.01) cells.

### MicroRNAs’ expression levels are differently modulated in response to cadmium treatments in PDAC cells

**Fig. 5:**
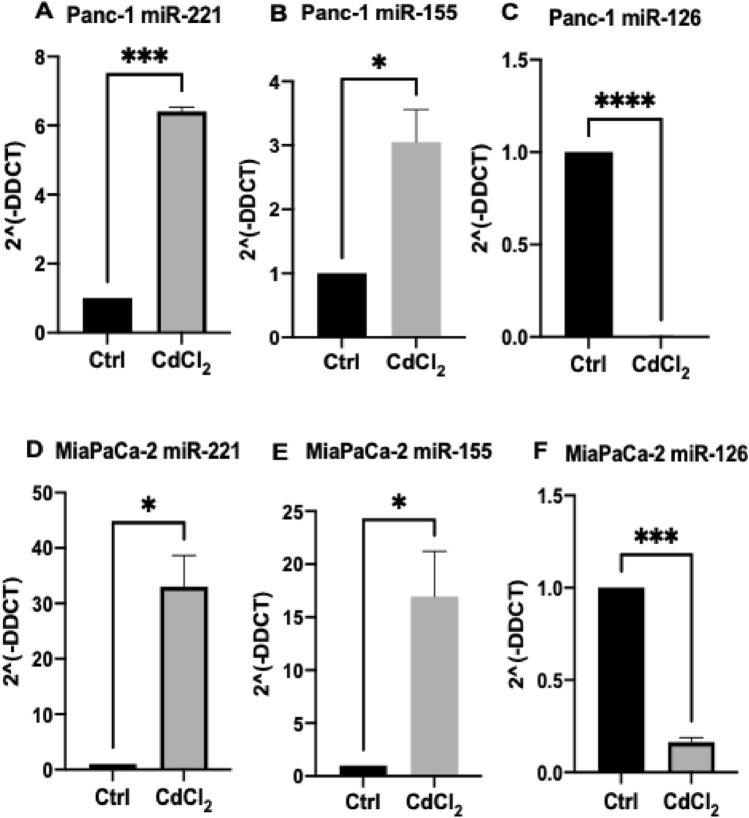
CdCl_2_ treatment-mediated effects on miR mRNA expression levels in PDAC cells. (**A**–**C)** Effects of CdCl_2_ exposure in the Panc-1 cell line: (**A**) miR-221 mRNA expression levels; (**B**) miR-155 mRNA expression levels; (**C**) miR-126 mRNA expression levels. (**D**–**F**) Effects of CdCl_2_ exposure in the MiaPaCa-2 cells (**D**) miR-221 mRNA expression levels; (**E**) miR-155 mRNA expression levels; (**F**) miR-126 mRNA expression levels. The column graphic represents the average of three replicates of RNA isolated from each cell line. Data normalised according to RNU6 expression by fold analysis (*n* = 3, *p* < *0.05*). Paired t test was used to examine the mRNA expression levels of the selected miRs following CdCl_2_ treatment compared with the non-treated PDAC cells (control cells). Exact *p* values are indicated (* *p* ≤ 0.05; ** *p* ≤ 0.01; *** *p* ≤ 0.001; **** *p* ≤ 0.0001); error bars indicate SD

Following the effect of the CdCl_2_ in apoptosis, we then continued to investigate its role in miR expressions. Based on our previous and others studies, specific miRs including miR-221, miR-155, and miR-126 are closely associated with the development of PDAC in different stages (Greither et al. 2010; Komoto et al. 2009; Su et al. 2013; Uysal-Onganer et al. 2021). Therefore, when assessing miRs expression levels (miR-155, miR-221, miR-126), significant expression changes were observed in response to CdCl_2_ treatment. Our current study showed that in the Panc-1 cell line, CdCl_2_ treatment significantly upregulated the mRNA expression levels of miR-221 by 65% (*n* = 3; *p* < 0.001; Fig. 5A) and miR-155 mRNA expression levels by 30% (*n* = 3; *p* < 0.05; Fig. 5B) compared to non-treated PDAC cells (control cells). Expression levels of miR-125 significantly downregulated by 100% (*n* = 3; *p* < 0.0001; Fig. 5C) in the same PDAC cell line. Similarly, our results found that in the MiaPaCa-2 cell line, mRNA expression levels of miR-221 (*n* = 3; *p* < 0.05; Fig. 5D) and miR-155 (*n* = 3; *p* < 0.05; Fig. 5E) were significantly increased by 33.5% and by 17%, respectively, following CdCl_2_ exposure, while mRNA expression levels of miR-126 were significantly reduced by 84% (*n* = 3; *p* < 0.001; Fig. 5F).

### Cadmium treatment differently affects Wnt-11, E-cadherin, Snail, and Zeb1 protein levels in Panc-1 and MiaPaCa-2 cells, following 14 days treatment

**Fig. 6:**
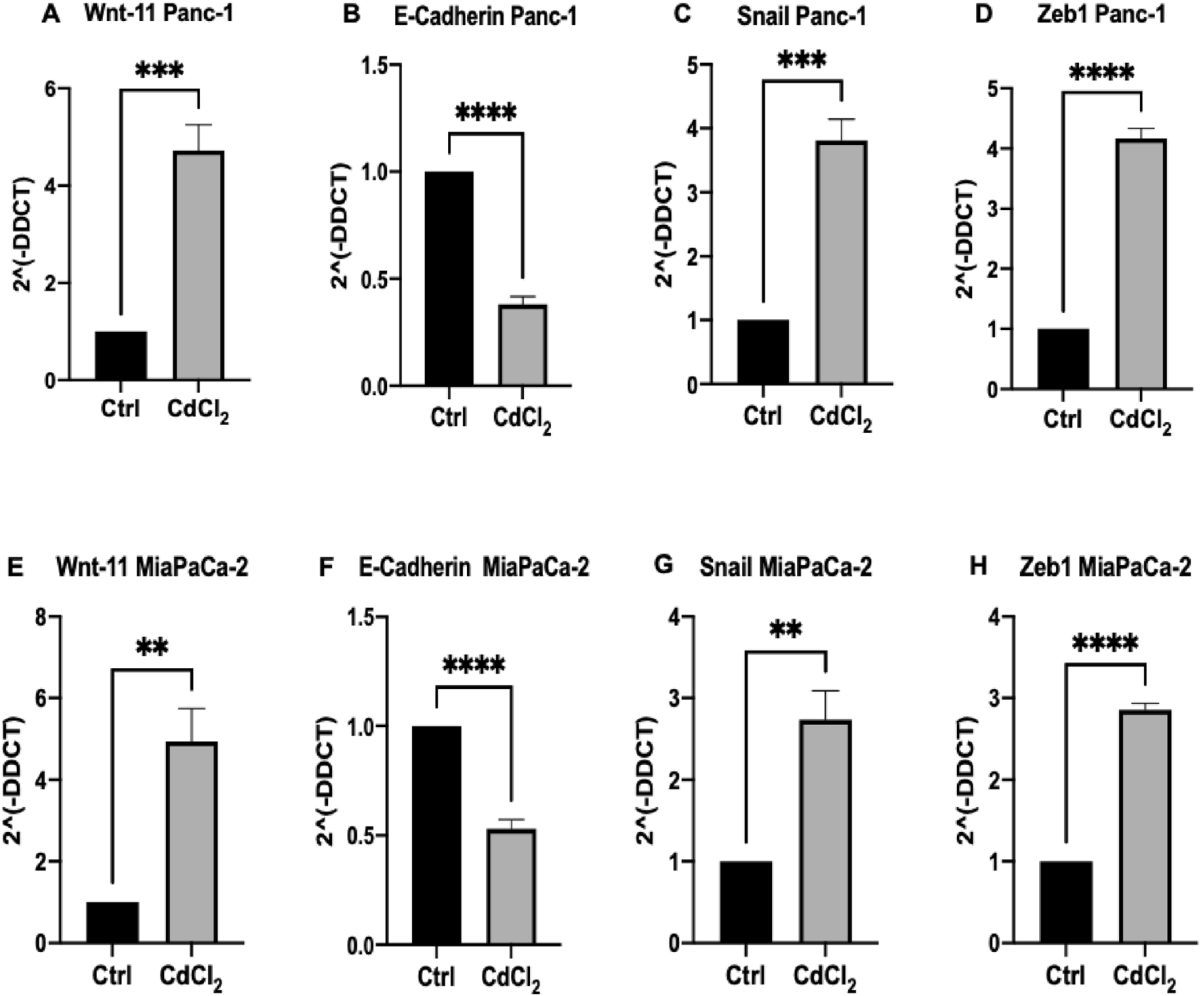
CdCl_2_ treatment-mediated effects on protein mRNA expression levels in PDAC cells. (**A**–**D)** Effects of CdCl_2_ exposure in the Panc-1 cell line: (**A**) Wnt-11 mRNA expression levels; (**B**) E-cadherin mRNA expression levels; (**C**) Snail mRNA expression levels; (**D**) Zeb1 mRNA expression levels. (**E**–**G**) Effects of CdCl_2_ exposure in the MiaPaCa-2 cells; (**D**) Wnt-11 mRNA expression levels; (**E**) Wnt-11 mRNA expression levels; (**F**) E-cadherin mRNA expression levels; (**G**) Snail mRNA expression levels; (**H**) Zeb1 mRNA expression levels. The column graphic represents the average of three replicates of RNA isolated from each cell line. Data normalised according to RPII expression by fold analysis (*n* = 3, *p* < *0.05*). Paired* t* tests were used to examine the mRNA expression levels of the selected proteins following CdCl_2_ treatment compared with the non-treated PDAC cells (control cells). Exact *p* values are indicated (* *p* ≤ 0.05; ** *p* ≤ 0.01; *** *p* ≤ 0.001; **** *p* ≤ 0.0001); error bars indicate SD

According to our previous and other reports, EMT markers play a significant role in different biological processes such as invasion and metastasis during PDAC development (Arumugam et al. 2009; Dart et al. 2019; Hotz et al. 2007; Iacobuzio-Donahue et al. 2009; Wang et al. 2018a, b). Therefore, based on our previous study, we extensively exposed both Panc-1 and MiaPaCa-2 PDAC cell lines with CdCl_2_ for 14 days, to further assess the protein levels of Wnt-11, E-cadherin, Snail, and Zeb1 by RT-qPCR (Djordjevic et al. 2019). In our current study, we found that in both PDAC cell lines, CdCl_2_ significantly upregulated Wnt-11, Snail, and Zeb1 expression levels and considerably reduced E-cadherin protein levels compared to non-treated PDAC cell lines (Fig. 6). Specifically, our results indicated that in the Panc-1 cell line, the mRNA expression levels of Wnt-11% (*n* = 3; *p* < 0.001; Fig. 6A), Snail (*n* = 3; *p* < 0.001*;* Fig. 6C), and Zeb1 (*n* = 3; *p* < 0.0001; Fig. 6D) were significantly increased by 47%, 38%, and 42%, respectively, following CdCl_2_ exposure compared with the non-treated Panc-1 cells (Panc-1 control cells), while mRNA expression levels of E-cadherin (*n* = 3; *p* < 0.0001; Fig. 6B) were significantly decreased by 62% in the same PDAC cell line. Similarly, in the MiaPaCa-2 cell line, the mRNA expression levels of Wnt-11 (*n* = 3; *p* < 0.01; Fig. 6E), Snail (*n* = 3; *p* < 0.01; Fig. 6G), and Zeb1 (*n* = 3; *p* < 0.0001; Fig. 6H) were significantly overexpressed by 50%, 28%, and 29%, respectively, following CdCl_2_ exposure compared with the non-treated MiaPaCa-2 cells (MiaPaCa-2 control cells), while mRNA expression levels of E-cadherin (*n* = 3; *p* < 0.0001; Fig. 6F) were significantly reduced by 47% in the same PDAC cell line.

## Discussion

The main outcome of our current study is that there is a strong correlation between CdCl_2_ exposure, miR expression, and protein expression associated with apoptosis.

Our study has methodically examined the effects of CdCl_2_ on the expression of multiple proteins associated with apoptotic pathways and as well as the expression of miRs that are involved in the cancer development and changes to apoptotic responses. We observed that CdCl_2_ exposure for 48 h exhibited a high degree of lethality on each of the cell lines with LC50 values 3–100 μM in normal growth media. In a reduced, or minimal, media, these values changed to 5–20 μM. The maximum lethality in all cell lines ranged from 70 to 85% at 1 mM CdCl_2_. We, and others, have shown that Cd itself is a weak pro-oxidant (Buha et al. 2017; Chen et al. 2011; Liu et al. 2009; Martinez-Zamudio and Ha 2011; Waisberg et al. 2003; Wallace et al. 2019). The ability to induce oxidative stress would be one mechanism in tumour development. We investigated multiple proteins associated with apoptosis, cell death, cellular repair, and growth regulation. Included in this group was cleaved caspase-3, phospho-Akt, PARP, PTEN, β-catenin, FOXO1, and p53. The major differences occurred between cell lines, with differing levels of expression. The main CdCl_2_ effects are observed with PTEN and FOXO1 expression with CdCl_2_ exposure resulting in increased expression of PTEN in both Panc-1 and Panc-10.05 cell lines. FOXO1 expression in Panc-10.05 was significantly reduced following exposure to CdCl_2_.

The miRs response in humans after Cd exposure is very complex, especially having in mind Cd ability to bioaccumulate in the body. So far, changes in miRs in terms of both up and downregulation were identified in ovarian granulosa cells (Wang et al. 2018a, b), kidneys (Pellegrini et al. 2016), prostate epithelial cells (Ngalame et al. 2016), hepatic cell lines (Urani et al. 2014), and bronchial epithelial cells (Liu et al. 2015). In our current study, we found that miRs expression levels have been significantly altered in PDAC cells lines treated with Cd from control cells. In particular, miR-221 and miR-155 expressions were considerably overexpressed in both Panc-1 and MiaPaca-2 PDAC cell lines treated with CdCl_2_, while miR-126 was significantly reduced.

As an oncogenic miR, miR-221 has been related to several epithelial cancers such as glioma (Lu et al. 2009; Siegel et al. 2014; Zhang et al. 2009, 2012), prostate carcinoma (Mercatelli et al. 2008), hepatocellular cancer (Gramantieri et al. 2009), and lung cancer (Garofalo et al. 2008), while the upregulation of this miR has been also linked to PDAC cell lines and tumour tissues in comparison to normal pancreatic tissues (Papaconstantinou et al. 2013). Other studies have highlighted that miR-221 not only upregulated in the MiaPaCa-2 cell line compared to normal human pancreatic ductal epithelium (Zhang et al. 2008), but also alterations in its expression levels can promote the metastatic propensity of PDAC cell lines (Xu et al. 2015). According to previous findings, upregulation of miR-221 is common in PDAC tissue samples compared to normal controls and, therefore, the expression of miR-221 in PDAC could be used for the discrimination of PDAC from benign PDAC tissue with specificity 93% (Bloomston et al. 2007). Importantly, when assessing the expression levels of miR-221 in the current study, miR-221 was identified to be significantly upregulated more in PDAC cells treated with CdCl_2_ compared with the non-treated PDAC cell, which highlighting that CdCl_2_ exposure increased the oncogenic property of miR-221 in PDAC cells. miR-155 is a further example of oncogenic miR, which is highly upregulated in several solid malignancies such as breast, colon, and thyroid cancers (Babar et al. 2011; Bakirtzi et al. 2011; Jiao et al. 2012; Nikiforova et al. 2008). Recent studies have indicated that miR-155 is one of the most oncogenic miR in PDAC (Bloomston et al. 2007; Szafranska et al. 2007), which is linked to poor prognosis of PDAC (Greither et al. 2010). miR-155 upregulation is also associated with PanIN-2 and PanIN-3 lesions compared to healthy pancreatic tissue (Ryu et al. 2010) and its upregulation was noticed in 80% of early pancreatic lesions (Ryu et al. 2010). In our current study, upregulated expression of miR-155 was found in both Panc-1 and MiaPaCa-2 cells treated with CdCl_2_ compared with the control cells_,_ which indicates a correlation between CdCl_2_ exposure and the oncogenic capability of miR-155. It has been also reported that the tumour suppressor miR-126 is correlated to numerous malignancies including lung, gastric, breast, and PDAC cancer (Feng et al. 2010; Sempere et al. 2010; Zhou et al. 2016). Specifically, reduced expression levels of miR-126 can lead to cellular migration and invasion through the inhibition of ADAM metallopeptidase domain 9 (*ADAM9*) target gene, which is commonly expressed in PDAC (Grutzmann 2004). Therefore, the reduced expression of miR-126 in PDAC has been reported in the previous studies, and this can be also associated with the results of our current study, which showed that mRNA expression levels of miR-126 was significantly decreased following CdCl_2_ exposure in the Panc-1 and MiaPaCa-2 cell lines.

In the current study, we also found that Cd regulates EMT and dysregulates the expression levels of mesenchymal markers including Wnt-11, E-cadherin, Snail, and Zeb1. Specifically, expression of Wnt-11, Snail, and Zeb1 was significantly overexpressed following the treatment with CdCl_2_, whereas E-cadherin was reduced in PDAC cell lines treated with CdCl_2_. miRs can promote not only proliferation and tumourigenesis in PDAC, but also to affect tumour microenvironment (Fathi et al. 2021). Specifically, hypoxia and dysregulations in the expression levels of EMT markers can be altered by several miRs (Lu et al. 2017). Consequently, carcinogenesis can be modulated by certain miRs and signalling cascades including Hedgehog, PTEN/Akt, Wnt, Signal transducer, and activator of transcription 3 (STAT3), ERK, JNK, TGF-β, and NF-κ (Fathi et al. 2021). EMT can be described as a morphologic cellular program, which represents the phenotypic transition from an epithelial to a mesenchymal state that is metastable (Wang et al. 2017). EMT is regulated by numerous complex modulatory networks such as epigenetic alterations, transcriptional control, which comprise EMT-inducing transcription factors (EMT-TFs) as Snail (Zinc finger protein SNAIL), Zeb (Zinc finger E-box-binding homeobox 1), and transcription regulators including miRs (Wang et al. 2017). Especially, miRs are considered as main regulators of EMT in several cancer types but also as novel early biomarkers in PDAC (Ali et al. 2015; Ballehaninna and Chamberlain 2013; Calatayud et al. 2017; Chang and Kundranda 2017; Dhayat et al. 2015; Gayral et al. 2014; Sethi et al. 2018; Winter et al. 2013). Specifically, miRs can regulate EMT through the control of their target messenger RNAs (mRNA) such as the E-cadherin transcriptional repressor ZEB-1 (Brabletz et al. 2011; Li et al. 2010; Krebs et al. 2017; Tang et al. 2016). For example, miR-200, miR-141, miR-200a, miR-200b, miR-200c, miR-429 (Gregory et al. 2008; Humphries and Yang 2015; Mongroo and Rustgi 2010), miR-34a (Ahn et al. 2012; Alemar et al. 2016; Tang et al. 2017), miR-148a (Feng et al. 2016; Peng et al. 2017; Tan et al. 2018, 2017), miR-203a (Jiang et al. 2017; McCubrey et al. 2016; Yang et al. 2017), and miR-655 (Harazono et al. 2013) act as EMT suppressors and negative regulators of metastatic predisposition of PDAC cells (Gregory et al. 2008; Peter et al. 2009), while miR-10b (Ouyang et al. 2014) and miR-197 (Hamada et al. 2013) strongly promote EMT. Particularly, miR-200 can inhibit the main regulators of EMT such as Zeb1 and Snail, which further results in the overexpression of E-cadherin levels (Gregory et al. 2008; Peter et al. 2009). Moreover, upregulation of miR-200c is linked not only to E-cadherin expression in resected human pancreatic tumour samples but also in better survival rates in comparison to patients with reduced miR-200c levels (Yu et al. 2010). Furthermore, Zeb1 can also suppress miR-200c and miR-141 transcription, which further contributes to the differentiation state of PDAC cells (Burk et al. 2008). Furthermore, miR-200 family can also inhibit Notch signalling, which is responsible for tissue homeostasis and is closely linked to EMT through the overexpression of Notch signalling and Zeb1 and decrease of miR-200 family in PDAC (Brabletz et al. 2011). A previous study showed that Zeb1 is a key target of miR-655, which is an EMT-suppressor miR that is correlated to favorable overall survival in PDAC patients (Harazono et al. 2013). Ahn et al., (2012) also indicated that miR-34a acts as a target of ZEB-1, which leads to the reduction of invasion and metastasis.

The downregulation of E-cadherin in PDAC patients has been widely described by the previous studies, which suggested that the loss of E-cadherin expression is detected in 43% of PDAC patients and associated with poor outcome and increased invasion and aggressiveness (Hong et al. 2011). The results of this study showed that the mRNA expression levels of E-cadherin were significantly reduced following the exposure of PDAC cells to CdCl2, which points out the importance of assessing the role of CdCl2 in EMT pathways during PDAC progression. Importantly, recent studies have suggested that Zeb1 and Snail overexpression is closely associated with PDAC progression and especially with inhibition of apoptosis and chemoresistance against gemcitabine in PDAC (Li et al. 2009; Vega et al. 2004; Yin et al. 2007). Based on this information, the results of the current study, which showed a significant upregulation of these EMT markers following CdCl_2_ exposure in PDAC cells, indicate the importance of CdCl_2_ in the regulation of these key tumourogenic PDAC EMT proteins. Additionally, other reports showed that Wnt-11 can enhance EMT, aggressiveness and migration in PDAC, which further affects the survival rates in PDAC patients (Dart et al. 2019). Specifically, Wnt-11 downregulation can promote not only EMT but also the expression levels of neuronal and stemness markers, which are linked to metastasis (Dart et al. 2019). In our current study, we particularly found that the mRNA expression levels of Wnt-11 were significantly overexpressed in Panc-1 and MiaPaCa-2 cells treated with CdCl_2_ compared to the control cells; which promote pilot insights into the role of CdCl_2_ in the Wnt-11 pathways. Conclusively, the Wnt family has been considered to be associated with TNM staging and especially with early stages of PDAC progression (Dart et al. 2019; Wang et al. 2018a, b; Wang et al. 2016), and plays a crucial role as regulator of stemness, EMT, and invasiveness of several cancer types comprising PDAC (Dart et al. 2019; Murillo-Garzón et al. 2018; Wei et al. 2014).

The existing evidence is clear, exposure to CdCl_2_, results in cellular damage that can lead to the development of PDAC. We have observed cellular effects consistent with changes in the expression of select miRs that are involved in tumour development. We have also observed changes in protein expression that may contribute to PDAC formation. Normal pancreatic function is maintained by normal functioning of various miRs. Multiple miRs have important roles in normal insulin function and sensitivity, diabetes, and PDAC development (Chakraborty et al. 2013). Examining the different pathways and potential overlap between cellular signalling mechanisms, relationships between miRs/gene/protein expression become evident. FOXO1 has been shown to inhibit the growth of tumours and can promote apoptosis (Mou et al. 2017). This is interesting considering CdCl_2_ exposure tended to reduce FOXO1 expression which would result in tumour growth. Data have shown that FOXO1, and other forkhead box transcription proteins, can increase the expression of pro-apoptotic genes and interfere with pathways utilizing Wnt/β-catenin, Akt, and PTEN, and is indirectly regulated by miR-221 (Lima et al. 2011; Mou et al. 2017). In breast cancer, miR-221 can work through both modulating the estrogen receptor gene, and FOXO1 expression (Mou et al. 2017). miR-221 regulates a target, Dickkopf 2, subsequently inhibiting the activation of the Wnt/β-catenin pathways (Chen et al. 2020). An inverse relationship between miR-221 and FOXO1 (downregulation of miR-221/upregulation of FOXO1) has been reported in ovarian cells in menopause (Wei et al. 2021). Overexpression of miR-221 can directly increase the growth and proliferation of PDAC cells by downregulating PTEN expression and upregulating phospho-Akt expression (Yang et al. 2016). The upregulation of miR-221 observed in our study did not translate to a reduction in PTEN and an increase in phospho-Akt. The tumour cell lines did show a slight increase in phospho-Akt expression following CdCl_2_ exposure, but no downregulation of PTEN expression. Downregulation of miR-221 will result in an increased expression of PTEN, as well as p27, p57, and PUMA which inhibits the growth and proliferation of PDAC cells (Park et al. 2009; Sarkar et al. 2013; Song et al. 2019). The regulation of p53 involves both positive and negative regulation, and the ability of p53 to regulate the expression of other miRs. Key miRs involved with p53 function are miR-34, miR-29, and miR-125 (Lima et al. 2011). Khakinezhad-Tehrani et al. (2021) reported that when miR-221 was suppressed, pro-apoptotic proteins such as p53 were overexpressed and the tumour suppressor, miR-34, was also overexpressed. The miR-221-mediated changes in p53 and miR-34 expression could then interact with FOXO protein expression and SIRT1 activity. p53 can stimulate miR-29, which inhibits PI3K and reducing expression and activity of Akt (Otsuka and Ochiya 2014). P53 directly and indirectly interacts with SIRT1 via miR-34, while SIRT1 can interact with FOXO proteins (Otsuka and Ochiya 2014; Yamakuchi 2012).

## Conclusion

PDAC is the most fatal malignancy, and therefore, it is crucial to develop a significant body of knowledge in the field of PDAC carcinogenesis. The role of environmental pollutants, such as Cd, in PDAC have been suggested but still not fully understood. This is the first research to indicate the role of miRs in response to Cd in PDAC, suggesting a significant correlation between miRs and Cd exposure during PDAC progression. These studies have also begun to establish a connection between apoptotic protein regulation, miRs, and Cd exposure in PDAC. Further studies are needed to investigate the precise role of miRs in PDAC progression as well as the role of Cd and other environmental pollutants in such processes. Furthermore, epigenetic data should be incorporated into risk assessments for Cd exposures improving our ability to predict outcomes and define more efficient prevention measures.

## Data Availability

Not applicable.

## Abbreviations

ADAM9: ADAM metallopeptidase domain 9
CA 19-9: Carbohydrate antigen 19-9
Cd: Cadmium
CdCl2: Cadmium chloride 2
DEVD peptide: Aspartyl-L-glutamyl-L-valyl-L-aspartic acid amide
EMT: Epithelial mesenchymal transition
EMT-TFs: EMT-inducing transcription factors
FBS: Foetal bovine serum
FOXO1: Forkhead box protein O1
HPNE: Human pancreatic nestin-expressing
HRP: Horseradish peroxidase
LC50: Lethal concentration 50%; concentration that will be lethal to half the population of cells
MAPK: Mitogen-activated protein kinase
microRNAs: MiRs/miRNAs
mRNA: Messenger RNA
NF-κβ: Nuclear factor kappa-light-chain-enhancer of activated B cells
P53: Tumour protein P53
PanIN: Pancreatic intraepithelial neoplasia
PARP: Poly (ADP-ribose) polymerase
PCa: Pancreatic cancer
PDAC: Pancreatic ductal adenocarcinoma
PDGF: Platelet-derived growth factor
PTEN: Phosphatase and tensin homolog
RPII: RNA polymerase II
SD: Standard deviation
SIRT1: Sirtuin 1
STAT3: Signal transducer and activator of transcription 3
TGF-β: Transforming growth factor beta
TNM: Tumour-node-metastasis
UTRs: Untranslated regions
